# An objective function exploiting suboptimal solutions in metabolic networks

**DOI:** 10.1186/1752-0509-7-98

**Published:** 2013-10-03

**Authors:** Edwin H Wintermute, Tami D Lieberman, Pamela A Silver

**Affiliations:** 1 Department of Systems Biology, Harvard Medical School, Boston, MA 02115, USA; 2 Wyss Institute for Biologically Inspired Engineering, Harvard University, Boston, MA, USA

**Keywords:** Metabolism, Variability, Metabolic flux analysis, Networks

## Abstract

**Background:**

Flux Balance Analysis is a theoretically elegant, computationally efficient, genome-scale approach to predicting biochemical reaction fluxes. Yet FBA models exhibit persistent mathematical degeneracy that generally limits their predictive power.

**Results:**

We propose a novel objective function for cellular metabolism that accounts for and exploits degeneracy in the metabolic network to improve flux predictions. In our model, regulation drives metabolism toward a region of flux space that allows nearly optimal growth. Metabolic mutants deviate minimally from this region, a function represented mathematically as a convex cone. Near-optimal flux configurations within this region are considered equally plausible and not subject to further optimizing regulation. Consistent with relaxed regulation near optimality, we find that the size of the near-optimal region predicts flux variability under experimental perturbation.

**Conclusion:**

Accounting for suboptimal solutions can improve the predictive power of metabolic FBA models. Because fluctuations of enzyme and metabolite levels are inevitable, tolerance for suboptimality may support a functionally robust metabolic network.

## Background

### Predicting metabolism with constraint-based models

Natural selection, acting within the laws of physics and chemistry, produces systems well adapted to local conditions. This basic evolutionary insight is formalized in constraint-based modeling. A biological system might be understood as the solution to a precisely formulated mathematical problem of optimization under constraint [1,2].

A constraint-based approach is often used to model metabolism in *E. coli*, for which nearly complete knowledge of the metabolic reaction network is available [3,4]. The exact stoichiometry of each biochemical reaction imposes a conservation-of-mass constraint that must hold in the steady state. In some cases thermodynamic, regulatory, or other constraints may be added to further restrict the possible metabolic reaction fluxes [5,6]. With the allowed flux space defined, common approaches select a flux vector that is optimal according to some hypothesized objective function [7]. This optimal solution represents an *a priori* prediction of all cellular metabolic fluxes.

The objective most often chosen for microbial models is the maximization of growth rate or yield, an approach we call simply Flux Balance Analysis (FBA). The utility of FBA relies on the assumption that growth rate approximates overall fitness and is the primary focus of selection. Numerous successful applications attest to the value and versatility of FBA for predicting growth rates in a variety of contexts [8-11]. However, standard FBA formulations face practical and principal limitations.

In practice, FBA generally cannot predict a unique rate for all fluxes. A solution which maximizes growth rate is typically mathematically degenerate, describing a region in flux space rather than a single point. Solution degeneracy is a well-described problem in systems in systems like metabolism which are flexible, internally redundant, and underdetermined by data [12]. Applications of FBA are therefore complicated when predictions are required for fluxes other than growth.

In principal, metabolism cannot function only to maximize growth rate. This is evidenced by deletions of metabolic genes in *B. subtilis*, some of which cause increased growth rates and biomass yields relative to wild type [13]. Selective pressure to increase growth rate must be balanced by other demands on metabolism. Investments in cellular maintenance, sensory apparatus, osmoregulation, intercellular communication, or motility may reduce growth rate while improving overall fitness. Finally, limitations of evolutionary time and genetic variability may mean that metabolism is simply imperfect, and not optimal for any objective [2]. Thus we can not necessarily exclude from consideration the many flux configurations that support, for example, 90% maximal growth.

We address both difficulties with a revision of the metabolic objective at the heart of genome-scale metabolic optimization models. We propose that microbial metabolism is better represented as a cloud of nearly optimal flux distributions, rather than a single ideal and fixed solution. Under this hypothesis, regulation drives cellular fluxes to within a degenerate optimal region. Solutions within this region are not further optimized and considered equally plausible. We present a mathematical formulation of this theory that preserves the properties of continuous and convex optimization that make standard FBA mathematically elegant and computationally quick.

Our model allows stronger predictions in some cases, despite strictly weakening the optimality assumption that grounds standard FBA. The Perturbed Solution Expected Under Degenerate Optimality (PSEUDO) outperforms comparable methods in predicting the redistribution of central carbon fluxes that occurs in metabolic mutants of *E. coli*. Our model attributes central metabolism with significant flexibility to negotiate the trade-off between optimizing growth rate and matching a target flux vector. This is particularly relevant for metabolic engineering applications when genetically modified cells must adapt to suboptimal flux profiles.

The success of the PSEUDO method suggests that degenerate optimality may be a basic organizing principle for metabolism. In support of this hypothesis, we will show that reported measures of flux variability correlate with the dimensions of the degenerate optimal region. More degenerate fluxes, which theoretically do not require precise values for fast growth, indeed exhibit more variation. Tolerance for numerous optimal and near-optimal flux configurations would be an asset to microbial metabolism, enabling robust growth in the face of perturbations to the network. A degenerate organization of metabolism may be an essential adaptation given fundamental physical constraints on the ability of cells to control their internal and external environments [14].

## Results and discussion

### Common approaches to genome-scale optimization: FBA and MOMA

**Figure 1:**
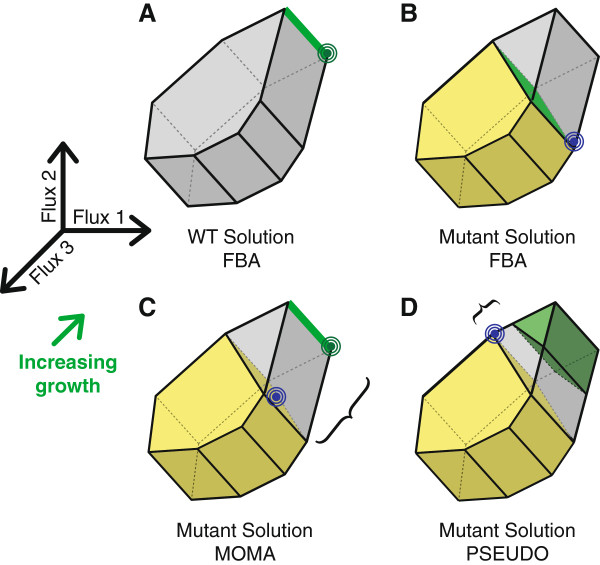
**FBA, MOMA and PSEUDO approaches to predicting metabolic fluxes. (A)** In FBA wild-type flux space is constrained to a polytope defined by thermodynamic and conservation-of-mass requirements. A linear objective describing cell growth, the green arrow, is maximized within this region. If the growth vector is perpendicular to a facet of the constrained polytope then a range of fluxes allow equally optimum growth, indicated by the heavy green edge. However, a linear programming solver can return only a single optimal point, the green target. **(B)** Mutations are represented as additional linear constraints that reduce the size of the allowed flux polytope. The yellow region represents the subset of wild-type fluxes allowed under a mutation. FBA finds a new optimum within this space as for the wild type. The green face represents a range of equally optimal mutant solutions. The blue target is a single point that a solver might return. **(C)** MOMA is an alternative approach for predicting mutant fluxes. The point in the mutant region, blue target, is found that minimizes the distance to a wild-type solution, green target. If FBA was used to generate the wild-type solution, then alternative optima may exist along the heavy green edge. **(D)** The PSEUDO strategy does not use FBA to select a wild-type flux vector. Instead we define a degenerate optimal region that contains all flux distributions capable of supporting near-maximal growth. A solution within the mutant region is found with minimum distance to this degenerate optimal region. Note that PSEUDO may select a point in mutant flux space different from the MOMA solution and closer to the growth-optimal region.

Figure 1 presents a geometric interpretation of the commonly used FBA and MOMA objective functions, contrasting them with the PSEUDO objective that we will describe below.

Standard FBA solves for a vector of metabolic fluxes within constrained flux space that maximizes cellular growth rate (Figure 1A). Because time is not represented in the model, predictions of growth rate or yield are treated identically. Mutations within the FBA framework are modeled as additional constraints that remove a region of the allowed flux space. For example, deletion of the *pgi* gene in *E. coli* eliminates phosphoglucose isomerase activity and constrains flux through that reaction to be zero. A mutant model is then re-solved to predict a new growth rate optimum (Figure 1B).

A popular alternative method for predicting mutant behavior is based on the Minimization of Metabolic Adjustment (MOMA) [15]. A mutant may not grow optimally if natural selection has not had a chance to act on the new genetic background. Instead, MOMA hypothesizes that a mutant will tend to approximate the wild-type state as closely as possible. Formally, a MOMA flux vector is found with minimum Euclidean distance to a single optimal wild-type profile, subject to the constraints of mutation (Figure 1C). This method therefore requires as input a unique optimal wild-type flux vector, which may be known from empirical measurements. In practice however, this point is often predicted with a standard FBA model.

Both FBA and MOMA use convex objective functions and convex constraints. Applications of these models can therefore access a powerful suite of convex programming algorithms [16]. A flux vector identified by convex programming is guaranteed to be unique, globally optimal, and can be computed in milliseconds. This is unlike most nonlinear optimization methods, which are computationally intensive and often can not guarantee a global optimum. The fast run times provided by convexity means that thousands of model variants can be rapidly re-solved in seconds on a conventional desktop. In metabolic engineering, for example, mutations can be screened combinatorially *in silico* for sets that improve production of a metabolite of interest.

### The problem of degeneracy in genome-scale models

Although solutions to FBA and MOMA problems are guaranteed to be globally optimum, they are not guaranteed to be unique. In practice, many different flux profiles allow equally optimum growth (Additional file 1: Figure S1 and Additional file 2: Figure S2). The problem of degeneracy is encountered frequently in the literature, and numerous attempts have been made to address it.

Degeneracy can be reduced by further constraining the model using known regulatory interactions [17], metabolite concentrations [18] or thermodynamic laws [19]. Previously measured flux rates, when available, are a particularly valuable guide for further predictions [15,20,21].

However, in many cases the additional information required to formulate these constraints is simply not available. Much of the power genome-scale methods is their potential to make predictions even in poorly characterized systems. Few metabolic flux measurements are available for most organisms, so here we have used only flux prediction techniques without flux measurements as inputs. Even in well known model organisms, regulatory interactions are only partially understood and imperfectly captured in a linear framework. In contrast, the approach we propose here uses only stoichiometric models and basic thermodynamic constraints, which can be inferred from any annotated genome sequence [22].

### PSEUDO: a new objective for microbial metabolism

A geometric interpretation of the PSEUDO method is presented in Figure 1D. We propose an objective function that explicitly accounts for a region of degenerate near-optimality. This region, **p**, is bounded as in a wild-type FBA model, with the additional constraint that it includes only flux configurations with nearly optimal growth. In this case, we set a threshold of at least 90% maximal growth rate on the vectors we will consider.

(1) $$\begin{array}{c} b_{L}\leq p\leq b_{U}\\ S\cdot p=\left[0\right]\\ p_{\mathrm{GROWTH}}\geq 0.90\cdot {\hat{f}}_{\mathrm{GROWTH}} \end{array}$$

The flux bounds **b**_*L*_ and **b**_*U*_ constrain fluxes that are known to be thermodynamically irreversible or that are limited by media inputs. The matrix **S** represents the biochemical stoichiometries of all metabolic reactions. The product **S**·**p** yields the net production or consumption rate of each metabolite in the system, necessarily **0** in the steady state. The maximum growth rate, ${\hat{f}}_{\mathrm{GROWTH}}$, derives from a standard FBA problem. Yet compared to a single-point FBA solution, the above is a more complete and conservative expression of what we can predict with confidence regarding growth-optimal fluxes. It expresses imperfection in the proposition that metabolism works only to maximize growth rate.

We then introduce a flux vector, **q**, representing a mutant version of the same organism. The **q** vector is not required to show near-optimal growth, but the mutation imposes the additional bounds, $b{'}_{U}$ and $b{'}_{L}$, on a subset of fluxes, **q**_*MUT*_. We propose that cellular regulation will push metabolism in the mutant, not towards a single optimal point as in MOMA, but towards a degenerate optimal region.

(2) $$\begin{array}{l} \begin{array}{l} \mathrm{minimize}:║p-q║\\ \mathrm{subject}\;\mathrm{to}: \end{array}\\ \begin{array}{l} \;b_{L}\leq p\leq b_{U}\\ p_{\mathrm{GROWTH}}\geq 0.90\cdot {\hat{f}}_{\mathrm{GROWTH}}\\ \;S\cdot p=\left[0\right] \end{array}\;\begin{array}{l} \;b_{L}\leq q\leq b_{U}\\ \;b{'}_{L}\leq \;q_{\mathrm{MUT}}\;\leq b{'}_{U}\\ \;S\cdot q=\left[0\right] \end{array}\; \end{array}$$

The above describes the geometric problem of finding the minimum distance between two polytopes: **p** representing the region of nearly optimal growth and **q** the space of possible fluxes limited by mutation. The point in **q** closest to the region **p** is the PSEUDO-predicted flux configuration for this metabolic mutant.

A PSEUDO solution will exist when the maximum mutant growth rate is less than the threshold set for the near-optimal region. Otherwise, the mutant and near-optimal flux polytopes overlap, describing a range of possible degenerate solutions. A minimum distance of 0 as a solution for (2) is immediately diagnostic for this degenerate case. A second form of degeneracy is possible in our model if the near-optimal and mutant polytopes align such that multiple solutions share the same non-zero Euclidean distance. This case is similar to classical FBA, in that an optimal objective value is defined but does not correspond to a unique solution.

As detailed in the Methods section, we can reformulate the objective ║p-q║ as a problem for either quadratic or conic convex programming. Therefore this formulation retains the desirable computational properties of FBA and MOMA, i.e. a guaranteed global optimum can be computed rapidly.

### PSEUDO growth predictions fall between FBA and MOMA predictions

**Figure 2:**
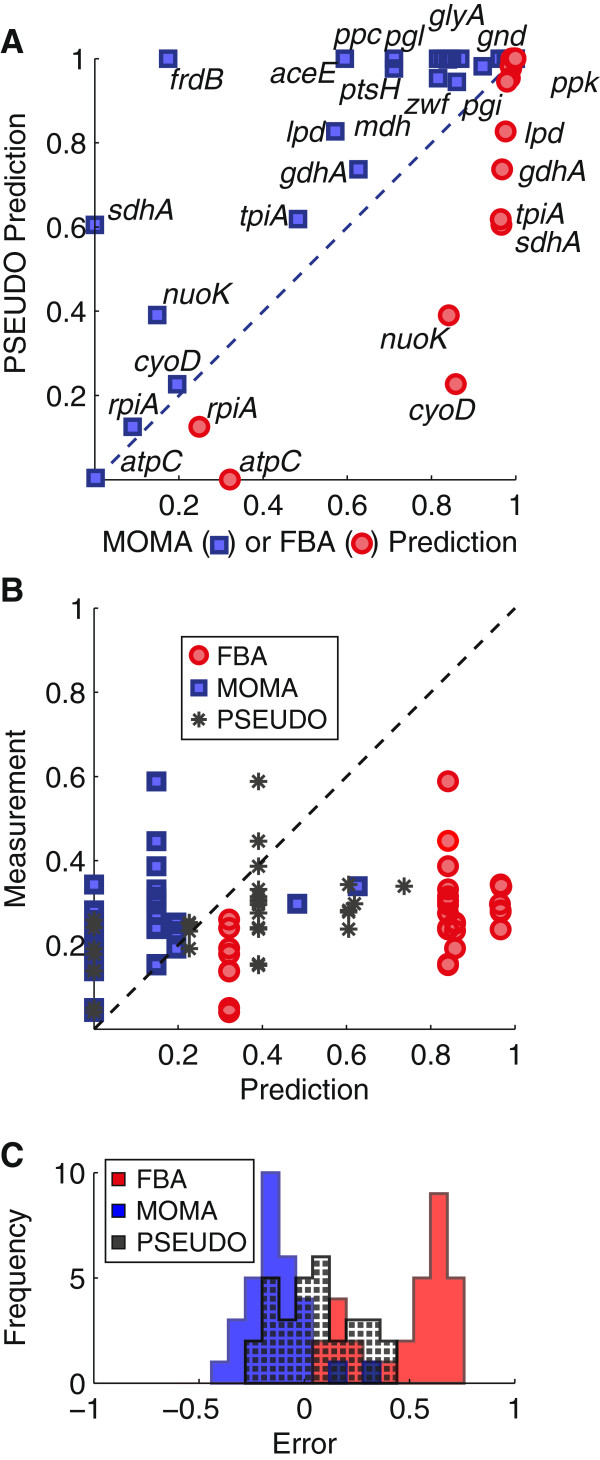
**PSEUDO growth predictions fall between FBA and MOMA.** Predicted and measured yields are both normalized to a maximum of 1, corresponding to wild-type growth in these conditions. **(A)** Yields are plotted for 41 mutants for which growth predictions differ by more than 5% among the three methods. The diagonal line indicates equal predictions in PSEUDO and other methods. Note that MOMA predictions are consistently above the diagonal, and FBA consistently below. **(B)** Compared with measured growth rate data, PSEUDO growth predictions produce a rank correlation *ρ* of 0.63 with yield data, compared to 0.45 for the FBA method and 0.42 for MOMA. **(C)** PSEUDO predictions show no systematic bias. The error is the difference between prediction and measurement. The mean of the error distribution for PSEUDO was 0.06 with a 95% confidence interval of [0.13, -0.01] by bootstrap resampling. FBA predictions exceeded growth rates by 0.48 on average [0.39, 0.54]. The mean MOMA prediction error was -0.13 [-0.18, -0.08]. While the PSEUDO errors were unbiased, FBA and MOMA predictions were systematic over- and underestimates, respectively.

Genome-scale optimization methods have a well-established utility for predicting growth rates of mutant strains under a variety of conditions. We compared growth rate predictions using the PSEUDO method to predictions from the FBA and MOMA techniques, both of which are commonly used for this purpose [23]. Predictions were compared to growth rates of *E. coli* deletion mutants from the Keio collection in defined glucose medium [24]. We compiled data for 795 mutant strains that could be represented in our model and for which growth rate data was available. The results of this comparison are shown in Figure 2.

The PSEUDO method was able to correctly predict the lethality phenotype for 88% of the deletion mutants examined. Results were comparable with FBA and MOMA (88% and 87%, respectively) and consistent with previous reports [4]. A deletion was predicted lethal if the growth rate was calculated to be less than 5% of the maximum. A deletion was empirically lethal if the measured OD yield was less than 5% of the maximum reported yield. The quantitative growth rate predictions made by the three methods were in general similar, with PSEUDO growth rates within 5% of the FBA optimum for 763 of the 795 mutants examined. Overall, FBA, MOMA and PSEUDO predictions correlated equally well with yield data (Spearman's *ρ* = 0.55, 0.54, 0.55, respectively). This general similarity was expected. Mutations which stoichiometrically block the production of biomass must be lethal by all three methods. Mutations in pathways that are not active in these media conditions will not affect growth predictions in any model.

However, PSEUDO growth predictions were found to differ by more than 5% from either FBA or MOMA predictions for 41 of the examined mutants (Figure 2A). This subset includes mutants bearing deletions of genes involved in glycolysis, the pentose phosphate pathway and the citric acid cycle. These disruptions in central carbon processing significantly impact cell fitness and require adaptations throughout the metabolic network, providing a challenging test case for predictive theories of metabolism. We therefore examined more closely behavior of our model in these cases.

Without exception, the PSEUDO growth prediction falls between those of FBA and MOMA. This pattern highlights an important feature of the PSEUDO objective function. FBA, by definition, calculates the highest possible growth rate consistent with thermodynamics and the conservation of mass. MOMA and PSEUDO growth predictions will both fall short of this maximum if there is a trade-off between matching the optimal growth rate and matching the rest of the wild-type flux vector. PSEUDO relaxes this trade-off by considering a range of targets within the degenerate optimal region and therefore PSEUDO growth rate predictions generally exceed those of MOMA.

The growth predictions that differ among the three models are plotted against measured growth data in Figure 2B. The mathematical requirement that PSEUDO predictions fall between FBA and MOMA correctly recapitulates the growth phenotypes of these strains. In all cases, a comparable rank correlation was found between predicted and observed growth rates (0.45, 0.42 and 0.63 for FBA, MOMA, PSEUDO respectively). However, we found the FBA predictions to consistently overestimate growth of these strains, while MOMA predictions were an underestimate in general. Figure 2C shows a histogram of prediction errors, the difference between measured and predicted yields for each method. The average PSEUDO prediction error was 0.06, and the mean of the error distribution was not significantly different from zero by either bootstrap resampling or ANOVA statistics (*p*-value: 0.2). In contrast, FBA systematically overestimated growth rates by 0.48 on average (*p*-value: 7.5·10^-9^). MOMA predictions were lower than predicted values by 0.13 on average, and were significantly biased to underestimation (*p*-value: 1.0·10^-8^).

### Precision and accuracy of PSEUDO flux predictions

We next sought to compare the biochemical flux predictions derived from our model to empirical flux measurements. The Metabolic flux rates of 31 central carbon reactions in 24 *E. coli* metabolic deletion mutants as determined by ^13^C-tracer experiments were reported by Ishii and [25]. We found that 12 of these mutants carried enzymatic deletions that could be treated within our genome-scale optimization framework, and that 27 fluxes showed measureable variation between strains. Accounting for occasional omissions in the data set, we were able to curate a total of 320 flux measurements against which to compare our predictions.

**Figure 3:**
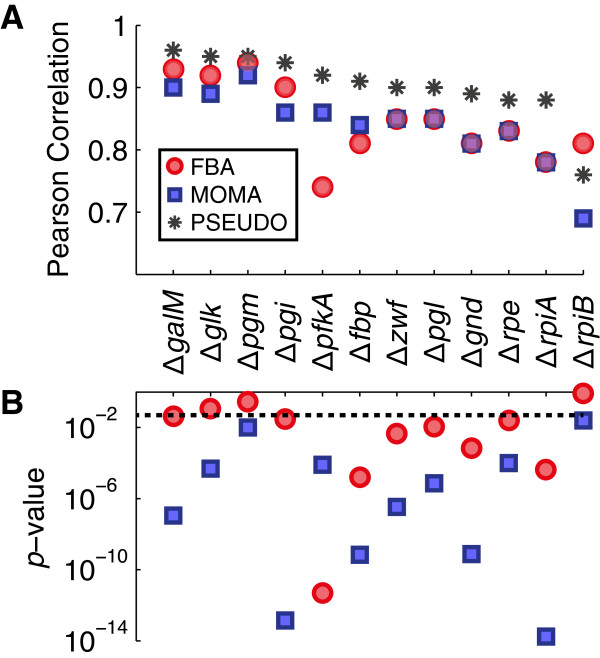
**Correlations of flux predictions by three methods.** Measured values of 31 fluxes from the Tomita data set were compared to predictions using the FBA, MOMA and PSEUDO objective functions. **(A)** Pearson correlations of flux predictions from the three methods for each of 12 metabolic deletion mutants. **(B)** Meng's Z-test was applied to the hypothesis that PSEUDO-derived correlation coefficients were higher than those derived using FBA (red circles) or MOMA (blue squares). The dotted line indicates a significance threshold of 0.05. PSEUDO significantly outperformed MOMA in every case and FBA in 9 of 12 cases. Exact correlation coefficients and *p*-values are reported in Additional file 3: Table S1.

Figure 3 compares measured fluxes values to predictions derived using the FBA, MOMA or PSEUDO objective functions. To facilitate comparison, both predicted and measured fluxes were normalized to the glucose uptake rate. Pearson correlation coefficients obtained for predictions within each of the 12 individual mutants ranged from 0.74 to 0.96. For 9 of the 12 strains, the PSEUDO method yielded a significantly higher correlation coefficient than either of the other two methods (p-value < 0.05). In one case, standard FBA produced the best predictions. In two cases, correlations from two or more methods were statistically equivalent. Statistical significance was assessed with Meng’s Z-test, which takes into account the high degree of correlation between the predictions from each method [26]. Similar significance results were obtained by bootstrap resampling. Exact correlation coefficients and p-values are reported in Additional file 3: Table S1.

**Figure 4:**
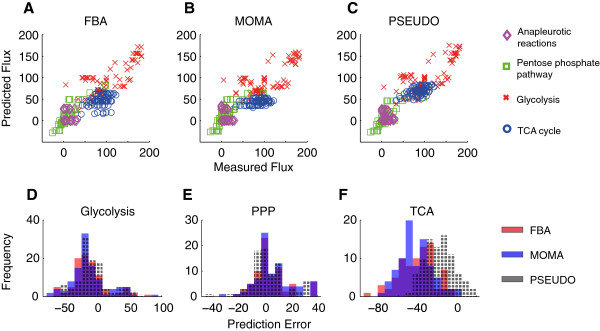
**Flux correlations and prediction errors for the PSEUDO method. (A, B, C)** Flux predictions derived using the FBA, MOMA and PSEUDO objective functions are plotted against 320 flux measurements taken from 12 different mutants in the Tomita data set. Flux values are reported as a percentage of the glucose uptake rate in each mutant. Symbols indicate the metabolic pathway associated with each flux. The FBA, MOMA and PSEUDO methods yielded Pearson correlation coefficients of 0.86, 0.84 and 0.91 respectively. The PSEUDO coefficient was significantly higher than that of the other two methods by both Meng's Z-test (*p*-value = 2.4·10^-12^) and bootstrap resampling (*p*-value < 1·10^-6^). **(D, E, F)** Histograms depicting the prediction errors for three metabolic pathways by three methods. While prediction errors were comparable among the three methods for fluxes belonging to the glycolysis and PPP class, errors in predicting TCA cycle fluxes were lower using the PSEUDO objective. Mean prediction errors of -41%, -42%, and -17% were obtained for FBA, MOMA and PSEUDO respectively. The reduced prediction errors using the PSEUDO method were significant by bootstrap resampling (*p*-value < 1·10^-6^).

For a global view of the metabolic behavior predicted by the FBA, MOMA and PSEUDO objective functions, we aggregated and examined predictions for all 320 measured fluxes across 12 mutants (Figure 4ABC). Across all 320 fluxes, PSEUDO was more predictive than FBA and MOMA (Pearson correlation coefficients of 0.86, 0.84 and 0.91 respectively for FBA, MOMA, and PSEUDO; *p*-value: 2.4·10^-12^, Meng’s Z-test). Because measured flux values span several orders of magnitude, we also compared predictions to data using rank correlation coefficients that are less influenced by numerical outliers. An overall Spearman rank correlation of 0.82, 0.80, and 0.87 was obtained with FBA, MOMA and PSEUDO respectively. The higher coefficient from PSEUDO was again significant by bootstrap resampling (*p*-value < 1·10^-6^).

Prediction accuracy was found to vary substantially for different pathways within central carbon metabolism. The histograms in Figure 4DEF compare prediction errors by each method in reactions belonging to glycolysis, the pentose phosphate cycle (PPP) and the tricarboxylic acid cycle (TCA) reactions. While prediction errors within glycolysis and the PPP were comparable among the 3 methods, the PSEUDO method produced significantly lower prediction errors within the TCA cycle. The FBA, MOMA and PSEUDO methods produced mean prediction errors of -41%, -42%, and -17% respectively. Flux variation within this class was also better predicted by PSEUDO. For TCA cycle reactions, FBA, MOMA and PSEUDO yielded Pearson correlations of 0.41, 0.38 and 0.60 respectively (*p*-value: 0.01). Thus, both the absolute level of TCA cycle flux and flux variations within the TCA cycle were better predicted by PSEUDO. Relatively high error in TCA cycle predictions when using growth as an objective in carbon-limited conditions has been reported in other models [15,27]. We found predictions within the TCA cycle to be both the most error-prone and the most revised under our model, being responsible for most of the improved performance. We found no other significant differences in predictions from the 3 methods in glycolysis, the PPP, anapleurotic, or secretion reactions.

### Sensitivity analysis of TCA cycle predictions

**Figure 5:**
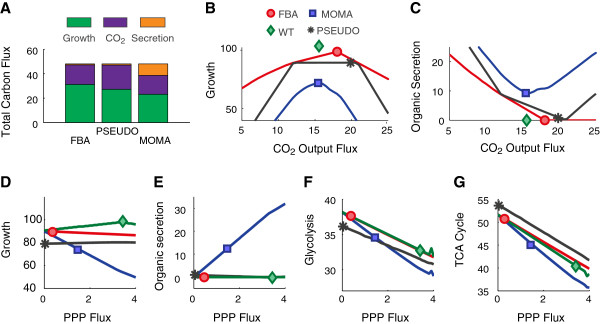
**Increased TCA cycle predictions using PSEUDO are partially explained by decreased growth, organic secretion and PPP fluxes. (A)** Carbon consumed as glucose may ultimately be converted into biomass, oxidized to CO_2_ or secreted as organic metabolites. For the *zwf* mutant, FBA predicts the most growth, MOMA predicts the most secretion and PSEUDO the most CO_2_ production. The molar flux is reported in units of mmol carbon gDW^-1^ hr^-1^. **(B, C)** Sensitivty analysis of growth and organic secretion with respect CO_2_ output. The WT model achieves optimal growth when CO_2_ output reaches 15 mmol gDW^-1^ hr^-1^. The FBA model of the *zwf* mutant attains 87% WT growth with a slightly higher optimal CO_2_ output. The MOMA model predicts a CO_2_ flux output for the *zwf* mutant similar to WT, with decreased growth and increased secretion. The PSEUDO objective identifies a wide range of CO_2_ output fluxes consistent with growth near 80% optimal. The high CO_2_ output selected by PSEUDO coincides with near-zero carbon secretion, similar to the WT. **(D)** PSEUDO predicts near-zero flux though the oxidative PPP in the *zwf* mutant, while both FBA and MOMA predict positive flux. Reducing oxidative PPP flux only marginally decreases growth (<1%) for FBA and MOMA predictions, while significantly increasing MOMA growth predictions. Growth is reported as percent WT. **(E)** Reducing PPP flux reduces organic secretion in the MOMA model, with no effect on secretion in other models. **(F, G)** Reduced PPP flux leads to increased glycolysis and TCA cycle fluxes. As the PPP flux approaches zero, the FBA, MOMA and WT predictions converge, suggesting this perturbation moves all three methods to PSEUDO-like solutions.

To understand why the PSEUDO objective function improves flux predictions for the TCA cycle, we chose to investigate central carbon metabolism in greater detail using the *zwf* mutant as a case study (Figure 5). Carbon consumed in the form of glucose may be converted to biomass, fully oxidized to CO_2_ or secreted as reduced organic metabolites. We reasoned that the metabolic strategy used to fulfill a given objective function would be reflected in the way carbon is partitioned among these three final forms.

We observed significant differences in the ultimate fate of carbon in the *zwf* mutant as predicted with the FBA, MOMA and PSEUDO objective functions (Figure 5A). The FBA model predicts the largest flux of carbon into biomass (63%, 48%, 56% and for FBA, MOMA, PSEUDO, respectively). This is consistent with the mathematical requirement that FBA identify the highest possible growth rate. The MOMA model predicts a significant amount of carbon secreted in the form of organic metabolites (0%, 20%, and 2% for FBA, MOMA, PSEUDO, respectively). The profile of secreted metabolites also varies among the models. While PSEUDO predicts mainly acetate secretion, MOMA predicts significant secretion of 33 diverse metabolites including urea, glutamate, leucine and tryptophan. Organic secretion is likely driven by the MOMA objective to match the WT flux profile. In effect, the MOMA attempts to produce metabolites at WT levels that cannot be completely consumed and partially secretes the difference.

The PSEUDO objective function predicts the highest CO_2_ output (37%, 32% and 42% for FBA, MOMA, PSEUDO respectively). This increase in total carbon oxidation is consistent with the higher TCA cycle flux predicted by PSEUDO. It may be explained by the relaxation in PSEUDO of strict optimality requirements used in the other methods. PSEUDO is not explicitly driven, like FBA, to optimize biomass production. Nor does it produce excess metabolites and secrete them, like MOMA. With less carbon flux dedicated to these objectives, the PSEUDO model retains more carbon to fully oxidize.

We next performed a sensitivity analysis to characterize the effect of CO_2_ output on the behavior of each model. We constrained the total CO_2_ output flux to a series of specific values near the WT optimum and re-solved the *zwf* mutant model to determine the fluxes to growth and organic secretion. The resulting plots reveal the trade-offs confronted in each objective function when assigning central metabolic fluxes near optimality (Figure 5BC).

In the FBA model, the *zwf* mutant reaches a maximum growth rate of 87% WT with a slightly increased CO_2_ output. The secretion flux approaches zero as growth attains a maximum, indicating that secreting organic carbon is costly to growth. The MOMA model predicts less growth and more secretion, but also exhibits a trade-off between the two. Any deviation of CO_2_ output levels from the WT optimum results in less growth and more secretion. In contrast, the PSEUDO solution is not found near a local growth maximum. Instead, the PSEUDO method identifies a range of CO_2_ output fluxes that are consistent with near-optimal growth. In the absence of a trade-off with growth, the PSEUDO objective is free to match other features of the WT flux vector. In this case, it selects a high CO_2_ flux that coincides with low secretion, similar to the WT.

To further characterize the assignment of TCA cycle fluxes in PSEUDO, we extended our analysis to examine the CO_2_ produced by the three main carbon-oxidizing pathways: Glycolysis, the PPP and the TCA cycle (Figure 5EFG). We observed a striking qualitative difference in the behavior of PSEUDO and the other objective functions. Uniquely, the PSEUDO objective function predicts that no CO_2_ is generated through the PPP. In contrast, FBA and MOMA predict that significant CO_2_ production through the oxidative reactions of the PPP (2.5% and 10% of the total for FBA and MOMA, respectively).

In fact, the *zwf* mutant shows no oxidative PPP activity in published observations [28]. During normal glucose-limited growth, the *zwf* gene product (together with *pgl*) supplies 6-phosphogluconate to the oxidative reactions of the PPP. In the *zwf* mutant, the FBA and MOMA models alternately supply this molecule through the action of glucose dehydrogenase and glucokinase. While *E. coli* is capable of oxidizing glucose directly to gluconate, the activity appears only under a narrow range of conditions not found in laboratory cultures [29].

As revealed by sensitivity analysis, small alterations the in the PPP flux had significant consequences for the predictive power of each model. Constraining the oxidative PPP flux to zero only slightly decreased the growth predictions for the FBA WT and *zwf* models (Figure 5D). This indicates that a zero-PPP solution exists near the WT optimum. However, neither FBA nor MOMA identified this solution. In the case of FBA, glucokinase activity bestows a small growth advantage, while MOMA is driven to match the high PPP flux of the WT.

When PPP flux was decreased, the MOMA model predicted significantly higher growth and lower secretion, indicating that a zero-PPP solution alleviated the trade-off between these two fluxes in MOMA. As PPP fluxes approach zero, glycolytic and TCA cycle fluxes increase and converge in all models, indicating that similar solutions were found by FBA, MOMA and PSEUDO under reduced PPP flux. We conclude that the low TCA cycle predictions in the MOMA and, to a lesser extent, the FBA model are partially a consequence of positive oxidative PPP fluxes. In contrast, PSEUDO approaches a solution with near-zero PPP flux, still consistent with near-optimal WT growth, and much less disruptive for the *zwf* metabolic network.

In summary, several features of the PSEUDO objective appear to contribute to higher and more accurate predictions of TCA cycle fluxes for the *zwf* mutant. The PSEUDO model grows less than FBA and secrete less than MOMA, leaving more carbon available to oxidize. PSEUDO is less constrained in matching growth rates, predicting equal growth from a wide range of possible CO_2_ output fluxes. Within this range, the PSEUDO objective selects a flux profile that matches key features of the WT. High CO_2_ and TCA fluxes are consistent with glycolytic fluxes and a secretion profile that resemble the WT.

In the case of the *zwf* mutant, the qualitative features of the PSEUDO prediction including the high growth rate, the increased CO_2_ production and zero oxidative PPP flux agree with published phenotypic observations [28]. In contrast, the FBA and MOMA predictions fail to reproduce one or more of these three qualitative phenotypic features.

### PSEUDO predictions are robust to the near-optimal growth threshold

We next sought to characterize the global behavior of the PSEUDO model as a function of the threshold that we use to define near-optimal growth. This value, set to 90% optimal growth in Equation 2, is a required input parameter for our model that has no equivalent in either FBA or MOMA. Mathematically, this represents a degree of uncertainty in the hypothesis that metabolism optimizes only growth rate. Biologically, this threshold could be understood as the point at which selection for growth is counterbalanced by other, unknown, selection pressures or by inherent noise. The results presented above were made using a threshold parameter of 90% optimal growth. This selection was guided by growth variability reported in the literature. For example, the reported growth rates of metabolic mutants of *B. subtilis* suggest that metabolism may be sub-optimal for growth at roughly this level under laboratory conditions [13].

**Figure 6:**
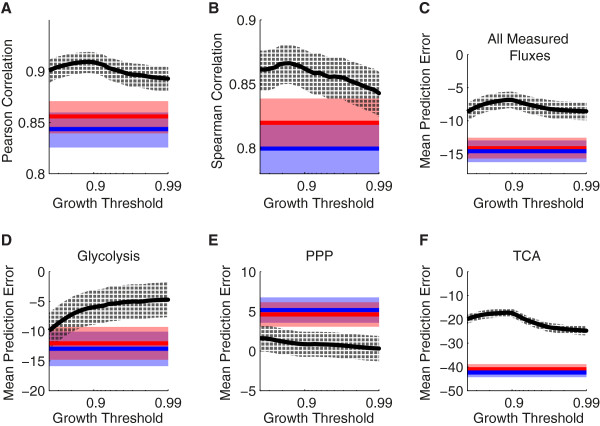
**PSEUDO predictions as a function of the near-optimal growth threshold.** Values derived using PSEUDO (black) are compared to values derived from FBA (red) and MOMA (blue). The PSEUDO method accepts a growth threshold input parameter that has no analogy in the other methods. This threshold defines a near-optimal flux space, and mutant flux profiles are determined that minimize the distance to this space. Biologically, this growth threshold could be interpreted as a region of relaxed regulation, within which selection for increased growth is balanced by other metabolic demands or by noise. The growth threshold parameter was allowed to vary from 80% to 99% WT maximal growth. **(A, B)** Pearson and Spearman correlation coefficients as a function of the near-optimal growth threshold. PSEUDO predictions reached a maximum using a growth threshold of 90%, but were generally robust to parameter variation. Error bars represent one standard error of the mean, calculated with Fisher's *z* transformation. **(C, D, E, F)** Mean flux prediction errors from each of the three methods as a function of the growth threshold parameter. Errors were calculated for all 320 fluxes curated from the Tomita data set, and for subsets of reactions belonging to glycolysis, the PPP, or the TCA cycle. Flux errors were generally insensitive to the chosen threshold. TCA cycle fluxes were both the most error-prone, and the most improved by PSEUDO. Error bars represent one standard error of the mean.

We found that PSEUDO predictions were remarkably stable as the near-optimal growth threshold was varied from 80-99%, as shown in Figure 6. Both Pearson and Spearman correlation values for PSEUDO predictions reached a maximum with the growth threshold set to 90%, and declined as near-optimal growth converged to maximum theoretical growth (Figure 6AB). We observed no qualitative differences in model behavior across this parameter range (Figure 6CDEF). This behavior is consistent with the convex shape of flux space. In a convex space, variability tends to increase rapidly for small deviations from optimality, then decelerate and plateau at moderate deviations [30-32]. Robustness with respect to the selected threshold is an important feature of the PSEUDO model, as this parameter may be difficult to measure in practice.

For very high or low values of the growth threshold, the practical application and biological interpretation of the PSEUDO model becomes more difficult. In practice, we were unable to compute solutions with threshold values higher than 99.9%, as extremely narrow range constraints on individual fluxes are known to challenge interior-point optimization solvers [33]. In this regime, we expect that PSEUDO predictions will become MOMA-like as the near-optimal region shrinks to become the optimal region. For low values of the growth threshold, the near-optimal region grows eventually to include the MOMA and FBA solutions and specific PSEUDO predictions are undefined.

### A cloud theory of metabolic regulation

The PSEUDO formulation includes a limit on the power of growth rate alone to determine metabolic behavior. This may represent simply a formal accounting for uncertainty, which is neglected in simpler models. Alternately, our objective function may be interpreted as expressing an organizational principal at work in metabolism. The metabolic network may not be perfectly adaptable in the service of growth, as imagined in FBA, nor strictly committed to a singular flux profile, as postulated by MOMA. The PSEUDO method mediates between these perspectives, attributing to metabolism an intermediate level of flexibility. Our model suggests that regulation drives metabolic fluxes to a certain range of values, but that fluctuations within that range are fitness-neutral and unregulated. On this hypothesis, we expect that regulation will allow fluxes to vary in proportion to the size of their near-optimal range.

To compare theoretical and observed flux variability, we first sought a properly normalized measure of the size of the degenerate optimal polytope in each dimension. While it is computationally infeasible to describe this space completely, [12,34], it is possible to estimate its shape probabilistically [32]. As described in the methods, we used a Monte Carlo sampling technique to generate a set of 3000 random points uniformly distributed within the degenerate optimal region [35,36]. These points allow an unbiased estimate of the distribution of values a given flux may attain without compromising near-optimal growth.

An ideal measure of flux variability *in vivo* would compile flux values from individual wild-type cells. Unfortunately, no such single-cell resolution dataset is currently available. Instead, we examined flux variability at the population level under two sources of genetic perturbation. The Tomita data set includes 31 fluxes measured in chemostat cultures of 24 deletion mutants for enzymes in central carbon processing [25]. Viable glycolysis and pentose phosphate pathway deletion mutants often exhibit substantially revised flux profiles and test the limits of metabolic plasticity. The Sauer data set measures 24 central carbon fluxes in 91 transcription factor deletion mutants [37]. These regulatory disruptions globally alter flux profiles while leaving the enzymatic network intact. We reasoned that these data sets together would allow us to characterize the tendency of individual fluxes to vary under perturbation.

**Figure 7:**
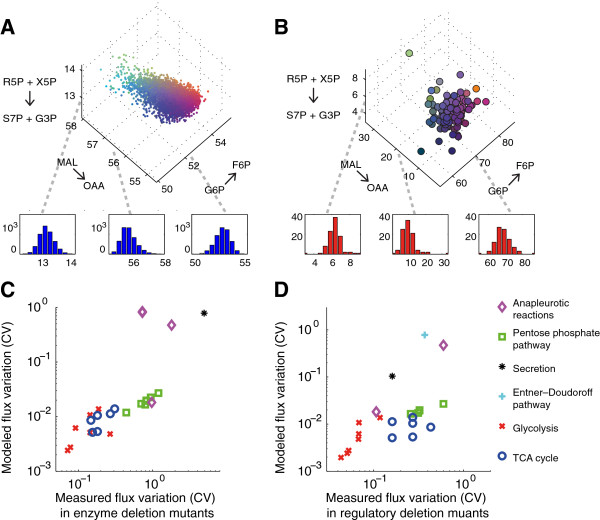
**A cloud theory for metabolic regulation.** The PSEUDO method hypothesizes the existence of a degenerate optimal region. Fluxes are regulated to approach this region, but allowed to vary freely within it. **(A)** Variability of fluxes within the degenerate optimal region. As described in the methods, we used Markov chain Monte Carlo sampling to produce 3000 randomly distributed fluxes, each consistent with at least 99% maximal growth. 3 specific fluxes are plotted on these axes, colored to emphasize their value in each dimension. Red, green and blue content corresponds to value in x, y and z respectively. Histograms present overall distributions of individual fluxes. **(B)** Variation in measured values for 24 fluxes across 91 transcription factor deletion mutants from the Sauer data set. Measured data is plotted similarly to the Monte Carlo generated data. **(C, D)** Computationally estimated flux variability within the degenerate optimal region correlates well with measured variability in both the Sauer **(C)** and Tomita **(D)** data sets. The coefficient of variation (CV) of the distributions is a normalized expression of both measured and predicted flux variability. Marker shapes are used to indicate the metabolic class of each flux. Metabolic fluxes that are only weakly coupled to biomass production can vary widely within the degenerate optimal region. The same fluxes are more likely to vary in under perturbation in published data sets.

Figure 7 compares our measures of theoretical and observed variation. For each flux in our data set, we compared the coefficient of variation (CV) derived from computational Monte Carlo sampling (Figure 7A) to the CV from published measurements (Figure 7B). Measured variability in both data sets was well matched by theoretical variability within the degenerate optimal region (Figure 7CD). The Sauer flux measurements produced a rank correlation of 0.72 (p-value: 6.7·10-5). For the Tomita data, Spearman's ρ was 0.87, (p-value: 5.6·10-9). Exact values for the estimated and measured variability of each flux are reported in Additional file 4: Table S2. The observed correlation between predicted degeneracy and measured variation supports a model in which metabolism may adopt many possible flux configurations without compromising growth rate.

## Conclusions

If the global behavior of metabolic regulation can be expressed mathematically as an objective function, then an optimization algorithm can identify the region of flux space that achieves this objective. The remarkable success of FBA, MOMA and other approaches in estimating metabolic behavior raises the tempting possibility that a truly general objective function may yet be found. To that end, recent work seeks to systematically compare the predictive performance of candidate metabolic objectives under diverse conditions [27,38,39].

Yet a single objective function may never fully capture the competing demands on a living biochemical network. A more versatile model of metabolism might be expressed in terms of multiple objectives and a formal framework for describing the tradeoffs among them [30,40,41]. However, combined objectives have limited predictive power without prior knowledge of how they will be balanced in a particular organism.

An alternative approach is to treat suboptimality as an inherent and irreducible feature of biology. Deviations from optimality may themselves be well ordered, and can reveal the action of novel selective forces and physiological constraints [2,42,43]. We have shown that variations in metabolic fluxes under perturbation can be largely explained if variation is constrained to be not growth-neutral, but nearly so. In general, metabolic systems biologists will be challenged to distinguish model errors due to a flawed objective function from variation which is suboptimal but constrained, or variation which is stochastic and does not satisfy any objective.

Finally, metabolism may simply be noisy. Metabolic flux space is large and high dimensional. It could remain so, even after all physical constraints have been satisfied and all functional objectives have been met. This would leave ample room for variation in flux rates between cells and within a cell over time. The metabolic behavior of a population may not be adequately described by any single flux profile. Seen from this perspective, suboptimal and degenerate solutions are not merely a technical inconvenience leading to non-unique predictions. They are rather an inherent and evolved property of metabolic organization.

We propose that a degenerate and variable biochemical flux profile could support a more robust cellular metabolism. Robustness, the capacity to perform an essential function despite perturbation, is a common property of biological systems [44]. Metabolic networks are already known to be replete with structural features that support robust function.

For example, the genes that code for many essential enzymes are carried in duplicate. Isoenzymes have the opportunity to diverge and specialize through evolution, but often remain capable of substituting for each other in the event that one becomes mutated or otherwise compromised [45,46]. This redundancy can extend from single genes to whole pathways, like the glyoxylate shunt, the methylglyoxal bypass, or the Entner–Doudoroff pathway, that provide alternate routes to key metabolic precursors [47].

Further, metabolic fluxes are typically robust to fluctuations in the abundance of the enzymes that catalyze them. While pathway fluxes can be controlled by the coordinated regulation of multiple enzymes, the total flux through a metabolic pathway rarely depends strongly on variations in the expression any single enzyme [48]. Most enzymes are produced in significant excess to the needs of a cell in standard conditions [49]. Perturbations that alter enzyme production are therefore unlikely to result in any given enzyme becoming flux-limiting. These sources of robustness may be particularly important given ubiquitous and substantial stochastic variation in protein copy number [50].

Degenerate optimality of the sort we have described would allow another level of functional redundancy. Even confronted with perturbations in enzyme function, regulation or copy number that destabilize intracellular fluxes, microbial growth would be unaffected so long as fluxes remain within the degenerate optimal region. Every cell must confront fundamental physical limits on its ability to regulate the internal environment [14]. A degenerate organization of metabolic fluxes would sidestep these limits, uncoupling growth rate from strict regulation which may be energetically costly or mechanistically impossible.

If selection and therefore regulation were relaxed with respect to growth rate, opportunities would emerge for selection to act on other features of the metabolic flux vector. For example, cells could anticipate a changing environment by minimizing the need to redirect fluxes under different metabolic conditions that are likely to appear [30]. Phenotypes within this open space could in principle be selected for any biochemical feature required by the local environment, without sacrificing near-optimal growth. Two bacteria may occupy the same metabolic niche and grow at the same rate, yet exhibit differently specialized flux profiles.

Strategic metabolic heterogeneity at the population level is already known in some systems. For example, *E. coli* populations under certain conditions display a bimodal distribution for expression of the lactose permease [51]. Individual cells can switch randomly between high or low enzyme levels, then propagate that state for a time scale of generations. A subset of the population is able to rapidly take up lactose, should it become available, while other subsets remain specialized for other carbon sources. This may be a way of hedging bets in unpredictable nutritive environments, without requiring each cell to prepare for all possible conditions [52,53]. Degenerate and growth-neutral variation in intracellular fluxes would allow another opportunity for adaptive bet hedging, as certain flux configurations may tend to better anticipate environmental changes. By tolerating growth-neutral variability, a more relaxed metabolic regulation would expand the range of accessible individual phenotypes.

Under this cloud theory of metabolic regulation, we expect flux magnitudes to exhibit substantial diversity at the single-cell level. This variation should occur even in clonal populations and uniform environments. It should correlate with the dimensions of the degenerate optimal flux region and have very little effect on growth rate. Phenotypic heterogeneity is increasingly seen to underlie complex emergent behaviors in genetic competence [54], antibiotic resistance [55], lysogeny [56], apoptosis [57], and stem cell induction [58]. Many other fundamental cellular properties are now known to vary substantially within populations [59-61]. We expect that the stochastic variations of metabolic fluxes within single cells, once measured, will reveal new modes of robust biological order.

## Methods

### FBA and MOMA

Standard FBA solves for a vector of metabolic fluxes, $\hat{f}$, that maximizes cellular growth rate, **f**_*GROWTH*_.

(3) $$\begin{array}{l} \begin{array}{l} \mathrm{maximize}:f_{\mathrm{GROWTH}}\\ \mathrm{subject}\;\mathrm{to}: \end{array}\\ \begin{array}{l} \;b_{L}\leq f\leq b_{U}\\ \;S\cdot f=\left[0\right] \end{array} \end{array}$$

The growth flux **f**_*GROWTH*_ represents metabolites leaving the system in the form of new biomass with a composition determined by measurement [62]. The matrix **S** represents the biochemical stoichiometries of all metabolic reactions. The product **S**·**f** yields the net production or consumption rate of each metabolite in the system, necessarily **0** in the steady state. The flux bounds **b**_*L*_ and **b**_*U*_ constrain fluxes that are known to be thermodynamically irreversible or that are limited by media inputs. Mutations in FBA are modeled by setting the appropriate flux bounds to zero.

The MOMA method [15] identifies a flux vector, **m**, with minimum Euclidean distance to an optimal wild-type solution, $\hat{f}$, subject to the constraints of mutation.

(4) minimize:║m‒f^║subjectto:bL≤m≤bUb'L≤mMUT≤b'US·m=0

The stoichiometric matrix, **S**, and the bounds, **b**, are defined as in (3). The modified bounds b’_*L*_ and b’_*U*_ are applied to the subset of fluxes eliminated by mutation, **m**_*MUT*_.

### Quadratic and conic formulations of PSEUDO

The PSEUDO method finds the minimum Euclidean distance between two high-dimensional flux polytopes, **p** and **q**. Constraints are added to place **p** within the region of degenerate optimality and impose a mutation on **q**.

(5) $$\begin{array}{l} \begin{array}{l} \mathrm{minimize}:║p-q║\\ \mathrm{subject}\;\mathrm{to}: \end{array}\\ \begin{array}{l} \;b_{L}\leq \;p\;\leq b_{U}\;b_{L}\leq \;q\;\leq b_{U}\\ p_{\mathrm{GROWTH}}\geq 0.9\cdot {\hat{f}}_{\mathrm{GROWTH}}\;b{'}_{L}\leq \;q_{\mathrm{MUT}}\;\leq b{'}_{U}\\ \;\left[\begin{array}{l} S\;0\\ 0\;S \end{array}\;\right]\cdot \left[\begin{array}{l} p\\ q \end{array}\right]=\left[\;0\;0\;\right] \end{array} \end{array}$$

Note that (5) is identical to (2) from the main text, except we have concatenated the conservation-of-mass constraints **S**·**p** = **0** and **S**·**q** = **0**. This form is standard for most solver algorithms.

In order to be analyzed with the powerful tools of convex programming, an optimization problem must be formulable with a convex objective under convex constraints [63]. The system of constraints above is composed only of linear inequalities, and is therefore convex. It remains only to provide a general convex formulation of the objective function:║p-q║. We first substitute the definition of a Euclidean distance between two vectors.

(6) $$\min\mathrm{imize}:\sqrt{{\sum_{i}\left(p_{i}-q_{i}\right)}^{2}}$$

Minimizing a positive radicand is equivalent to minimizing the root, so we neglect the radical. The objective then expands to:

(7) $$\min\mathrm{imize}:\sum_{i}p_{i}^{2}-2p_{i}q_{i}-q_{i}^{2}$$

Finally we reformulate the above as a linear algebraic expression, with **I** representing the identity matrix:

(8) $$\mathrm{minimize}:\;\left[p\;q\right]\;\cdot \;\left[\;\begin{array}{l} \;I\;‒I\;\\ ‒I\;I \end{array}\;\right]\;\cdot \;\left[\;\begin{array}{c} p\\ q \end{array}\;\right]$$

The square matrix in the above expression is symmetric and positive semidefinite, with eigenvalues of only 2 or 0. This is sufficient to guarantee convexity and polynomial solvability [64].

It is also possible to formulate PSEUDO as conic optimization problem. Conic optimization is among the most general forms of nonlinear convex optimization treated by commercial solvers. In this case we add a vector **x** to our variable set with the linear constraint that x_i_ = p_i_ - q_i_. The **x** vector and a new variable, *z*_*DIST*_, are further constrained to lie within a convex quadratic cone *C* such that *z*_*DIST*_ is at least the Euclidean distance between **p** and **q**. Minimizing *z*_*DIST*_ under these constraints produces the shortest distance from **p** to **q**.

(9) $$\begin{array}{l} \begin{array}{l} \mathrm{minimize}:z_{\mathrm{DIST}}\\ \mathrm{subject}\;\mathrm{to}: \end{array}\\ \begin{array}{l} \;b_{L}\leq \;p\;\leq b_{U}\;b_{L}\leq \;q\;\leq b_{U}\\ p_{\mathrm{GROWTH}}\geq \;0.99\cdot {\hat{f}}_{\mathrm{GROWTH}}\;b{'}_{L}\leq \;q_{\mathrm{MUT}}\;\leq b{'}_{U}\\ \begin{array}{l} \left[\begin{array}{c} S\;0\;0\\ 0\;S\;0\\ I\;‒I\;‒I \end{array},\;\right]\;\cdot \left[\begin{array}{c} p\\ q\\ x \end{array}\right]=\;\left[0\;0\;0\right] \end{array}\\ \left[\;x\;z_{\mathrm{DIST}}\right]\;\in C\;:=\;\left\{z_{\mathrm{DIST}}\geq \sqrt{\sum_{i}x_{i}^{2}}\right\} \end{array} \end{array}$$

### Linear, quadratic, and convex programming

All convex programming was implemented in MATLAB and solved using MOSEK optimization software.

All *E. coli* metabolic simulations were implemented using the iAF1260 model, incorporating stoichiometric and thermodynamic but not regulatory constraints [4]. Media parameters were defined minimal glucose media, using standards set by the model authors. Glucose availability was set to 8 mmol per gram dry weight (gDw) per hour, oxygen to 18.5 mmol gDw^-1^ hr^-1^, with ammonia, phosphate, sulfate and trace minerals available in excess. Growth-associated ATP consumption was set to 59.81 mmol gDw^-1^, with 8.39 mmol ATP gDW^-1^ hr^-1^ required for growth-independent cellular maintenance. Model conditions correspond to a standard media formulation as used in the experimental data set [25]. Flux values were normalized to the glucose uptake rate of each mutant, and are reported as a percentage of this value.

The MOMA solver requires a wild-type flux distribution as input. In all cases, this was calculated as an FBA prediction for the wild type strain with the same media conditions and biomass composition. Empirically measured fluxes were not an input to any solver.

A secondary objective function was used with the FBA and MOMA techniques to minimize the 1-norm of a growth-optimal solution vector. For the FBA method, this secondary objective was applied directly to the mutant solution. For MOMA, the secondary optimization was applied to the FBA-derived wild-type flux vector.

Unless otherwise indicated, the PSEUDO solutions reported were calculated using a growth threshold parameter of 90% the WT maximum, as calculated with standard FBA.

Sensitivity analysis was performed by constraining the fluxes corresponding to CO_2_ output or phosphogluconate dehydrogenase to a range of exact values and re-solving each model. Molar carbon secretion fluxes were obtained as the sum of all output fluxes weighted by the number of carbon atoms in each output molecule.

### Uniform sampling from flux space

Random flux configurations were generated by Monte Carlo sampling [35].We used an artificially centered hit-and-run algorithm to produce a series of points within the degenerate optimal flux polytope [36]. As this series is extended, it asymptotically approaches a uniform random distribution.

Briefly, a random set of initial points was generated within the degenerate optimal region by solving a linear programming objective to maximize a random vector chosen on the unit sphere. These solutions sample the extreme vertices. The hit-and run algorithm is initialized from the center of these extrema, which by the definition of convexity must also lie within the degenerate optimal region. A direction is chosen as the difference between a random extreme point and the center point. Then a line is drawn in this direction from the initial point to the region boundary and a new point is selected randomly from this line. After a sufficiently large number of such movements, the point reached is as drawn from a uniform distribution.

Following Bordbar *et al*., we define a mixed fraction for a set of points that allowed us to determine when our algorithm has reached an approximately uniform distribution [35]. We partition the initial points into two sets along the median value of any flux. The mixed fraction is defined as the number of points which cross the line as the algorithm progresses. The mixed fraction approaches 50% asymptotically, with the algorithm terminated at a value of 53%.

## Competing interests

The authors declare that they have no competing interests.

## Authors’ contributions

EW and TL constructed the theory and derived the model predictions. EW, TL and PS analyzed the results and drafted the manuscript. All authors read and approved the final manuscript.

## Supplementary Material

Additional file 1: Figure S1 Degeneracy in Metabolic Flux Analysis. (A, B) Two alternate flux distributions in central carbon metabolism that support equally optimal growth. Configuration A uses the pentose phosphate cycle to oxidize glucose. Configuration B runs the pentose phosphate cycle in reverse only as a source of precursor metabolites. While A produces more NADPH, B produces more pyruvate for the TCA cycle. Both options identically supply the ATP, reducing equivalents and carbon skeletons needed for growth. Click here for file

Additional file 2: Figure S2 Fluxes vary widely within the degenerate optimal range. As described in the main text, the degenerate range is the gap between the minimum and maximum possible values for each flux that support above a threshold near the maximum value. (A, B, C) The degenerate range in these three panels is calculated under the constraint of 99.9%, 99% and 95% maximal growth, respectively. The flux magnitude is calculated for the wild type system using FBA. Fluxes above the grey dotted line can vary by more than 100 times their predicted value without compromising growth. Fluxes on the red dotted line can vary by exactly 1%. Infinite flux variability is possible for fluxes in futile cycles. Zero flux variability indicates a metabolic reaction that cannot occur under these media conditions. Example enzymes from various metabolic pathways are indicated. (D) Flux variability for selected reaction classes. For nonzero fluxes, fold degeneracy is the degenerate range divided by the FBA-predicted flux magnitude. The median fold degeneracy is plotted for each class, with bars indicating the 10th and 90th quantiles. Click here for file

Additional file 3: Table S1 Correlations of flux predictions by three methods. 31 Measured flux values from the Tomita data set were compared to predictions using the FBA, MOMA and PSEUDO objective functions. Reported values are Pearson correlation coefficients. Meng's Z-test was used to test the hypothesis that the PSEUDO-derived correlations were higher than those from each other method. Similar significance results were obtained by bootstrap resampling. Entries in bold and marked with an asterix indicate that the PSEUDO method was not more predictive than both other methods at a *p*-value less than 0.05. Click here for file

Additional file 4: Table S2 Flux variability under perturbation and the shape of the degenerate optimal region. As described in the main text, flux measurements were collected from two published data sets. The Tomita data set consists of 31 fluxes measured in 24 deletion mutants for central carbon processing enzymes. The Sauer data set includes 24 fluxes from 91 deletion mutants for regulatory transcription factors. The standard deviation, σ, divided by the mean flux value yields the coefficient of variation, CV, a unitless measure of flux variability. The Markov Chain Monte Carlo method was used to generate 3000 random points uniformly distributed within the degenerate optimal region of the iAF1260 FBA model of E. coli. Each of these points corresponds to a possible flux profile sustaining growth at at least 90% of the theoretical maximum. The mean and standard deviation of the MCMC-generated points yield an estimated CV representing the variation possible for each flux while still maintaining near-optimal growth. As depicted in Figure 7 of the main text, simulated flux variability correlates with measured variability from both data sets. This suggests a model in which relatively tight regulation is applied only to fluxes that strongly impact cell growth. Inversely, substantial variation is tolerated in fluxes with less impact on growth rate. Click here for file
