# Supplementary figures and images for: An objective function exploiting suboptimal solutions in metabolic networks
Source: BMC Syst Biol. 2013 Oct 3;7:98. doi: 10.1186/1752-0509-7-98 (PMC4016239; doi:10.1186/1752-0509-7-98)

**A**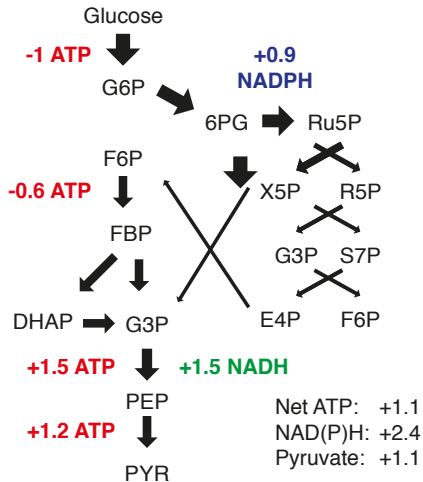**B**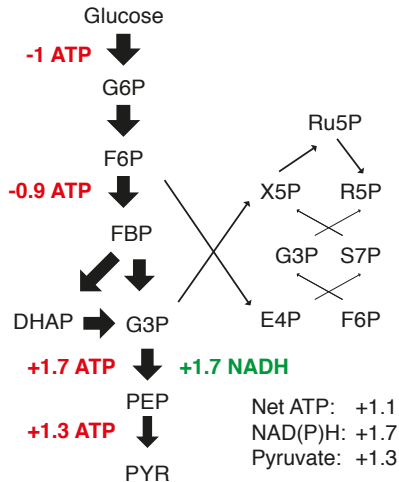

Supplement: Additional file 1: Figure S1 — Degeneracy in Metabolic Flux Analysis. (A, B) Two alternate flux distributions in central carbon metabolism that support equally optimal growth. Configuration A uses the pentose phosphate cycle to oxidize glucose. Configuration B runs the pentose phosphate cycle in reverse only as a source of precursor metabolites. While A produces more NADPH, B produces more pyruvate for the TCA cycle. Both options identically supply the ATP, reducing equivalents and carbon skeletons needed for growth. [file 1752-0509-7-98-S1.pdf]
