# Supplementary material for: An objective function exploiting suboptimal solutions in metabolic networks
Source: BMC Syst Biol. 2013 Oct 3;7:98. doi: 10.1186/1752-0509-7-98 (PMC4016239; doi:10.1186/1752-0509-7-98)
Supplement: Additional file 2: Figure S2 — Fluxes vary widely within the degenerate optimal range. As described in the main text, the degenerate range is the gap between the minimum and maximum possible values for each flux that support above a threshold near the maximum value. (A, B, C) The degenerate range in these three panels is calculated under the constraint of 99.9%, 99% and 95% maximal growth, respectively. The flux magnitude is calculated for the wild type system using FBA. Fluxes above the grey dotted line can vary by more than 100 times their predicted value without compromising growth. Fluxes on the red dotted line can vary by exactly 1%. Infinite flux variability is possible for fluxes in futile cycles. Zero flux variability indicates a metabolic reaction that cannot occur under these media conditions. Example enzymes from various metabolic pathways are indicated. (D) Flux variability for selected reaction classes. For nonzero fluxes, fold degeneracy is the degenerate range divided by the FBA-predicted flux magnitude. The median fold degeneracy is plotted for each class, with bars indicating the 10th and 90th quantiles. [file 1752-0509-7-98-S2.pdf]

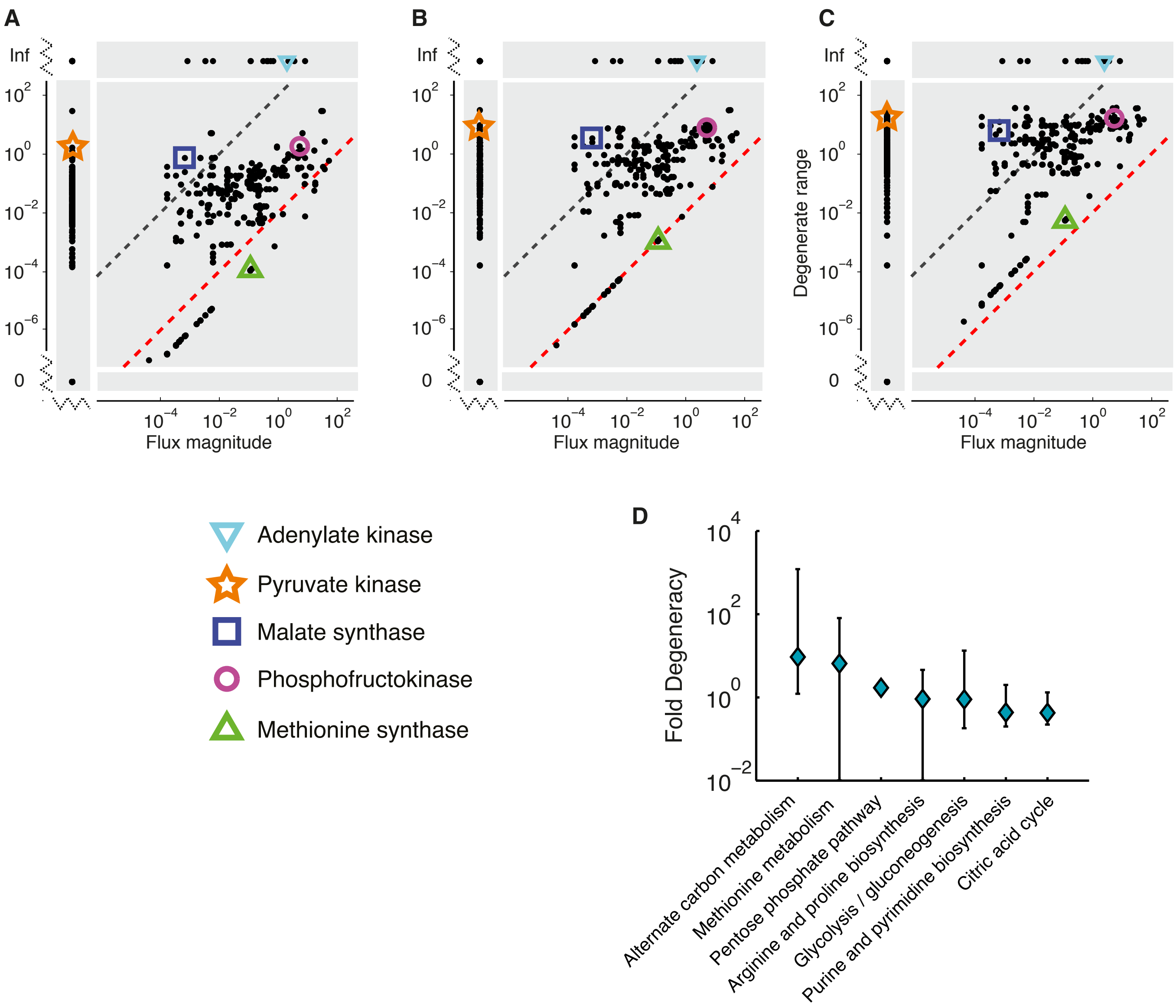

A
B
C
D
Inf
10^2
10^0
10^{-2}
10^{-4}
10^{-6}
0
10^{-4}
10^{-2}
10^0
10^2
Flux magnitude
Degenerate range
Adenylate kinase
Pyruvate kinase
Malate synthase
Phosphofructokinase
Methionine synthase
Fold Degeneracy
10^4
10^2
10^0
10^{-2}
Alternate carbon metabolism
Methionine metabolism
Pentose phosphate pathway
Arginine and proline biosynthesis
Glycolysis / gluconeogenesis
Purine and pyrimidine biosynthesis
Citric acid cycle
