# Supplementary material for: An objective function exploiting suboptimal solutions in metabolic networks
Source: BMC Syst Biol. 2013 Oct 3;7:98. doi: 10.1186/1752-0509-7-98 (PMC4016239; doi:10.1186/1752-0509-7-98)
Supplement: Additional file 3: Table S1 — Correlations of flux predictions by three methods. 31 Measured flux values from the Tomita data set were compared to predictions using the FBA, MOMA and PSEUDO objective functions. Reported values are Pearson correlation coefficients. Meng's Z-test was used to test the hypothesis that the PSEUDO-derived correlations were higher than those from each other method. Similar significance results were obtained by bootstrap resampling. Entries in bold and marked with an asterix indicate that the PSEUDO method was not more predictive than both other methods at a p-value less than 0.05. [file 1752-0509-7-98-S3.doc]

| **Mutant Strain** |  | **FBA** | **MOMA** | **PSEUDO** |  | ***p*-value:**  **PSEUDO**  **> FBA** | ***p*-value:**  **PSEUDO**  **> MOMA** |
| --- | --- | --- | --- | --- | --- | --- | --- |
| ∆*galM* |  | 0.85 | 0.85 | 0.90 |  | 4.5 · 10^-3^ | 3.4 · 10^-7^ |
| ∆*glk* |  | 0.81 | 0.81 | 0.89 |  | 6.6 · 10^-4^ | 7.5 · 10^-10^ |
| ∆*pgm* |  | 0.81 | 0.84 | 0.91 |  | 1.6 · 10^-5^ | 7.0 · 10^-10^ |
| **∆*pgi* *** |  | **0.81** | **0.69** | **0.76** |  | **8.6 · 10^-1^** | **2.5 · 10^-2^** |
| ∆*pfkA* |  | 0.85 | 0.85 | 0.90 |  | 1.1 · 10^-2^ | 7.2 · 10^-6^ |
| ∆*fbp* |  | 0.83 | 0.83 | 0.88 |  | 2.5 · 10^-2^ | 1.0 · 10^-4^ |
| **∆*zwf* *** |  | **0.92** | **0.89** | **0.95** |  | **1.2 · 10^-1^** | **4.9 · 10^-5^** |
| **∆*pgl* *** |  | **0.94** | **0.92** | **0.95** |  | **2.7 · 10^-1^** | **1.0 · 10^-2^** |
| ∆*gnd* |  | 0.93 | 0.90 | 0.96 |  | 4.4 · 10^-2^ | 1.1 · 10^-7^ |
| ∆*rpe* |  | 0.90 | 0.86 | 0.94 |  | 2.9 · 10^-2^ | 1.4 · 1 ^-13^ |
| ­∆*rpiA* |  | 0.74 | 0.86 | 0.92 |  | 5.0 · 10^-12^ | 8.1 · 10^-5^ |
| ∆*rpiB* |  | 0.78 | 0.78 | 0.88 |  | 4.3 · 10^-5^ | 1.7· 10^-14^ |

**Supplementary Table S1. Correlations of flux predictions by three methods.** 31 Measured flux values from the Tomita data set were compared to predictions using the FBA, MOMA and PSEUDO objective functions. Reported values are Pearson correlation coefficients. Meng's Z-test was used to test the hypothesis that the PSEUDO-derived correlations were higher than those from each other method. Similar significance results were obtained by bootstrap resampling. Entries in bold and marked with an asterix indicate that the PSEUDO method was not more predictive than both other methods at a *p*-value less than 0.05.
